# Preparation and Evaluation of Carbamazepine Solid Lipid Nanoparticle for Alleviating Seizure Activity in Pentylenetetrazole-Kindled Mice

**DOI:** 10.3390/molecules24213971

**Published:** 2019-11-02

**Authors:** Mona Qushawy, Kousalya Prabahar, Mohammed Abd-Alhaseeb, Shady Swidan, Ali Nasr

**Affiliations:** 1 Department of Pharmaceutics, Faculty of Pharmacy, University of Tabuk, Tabuk 471, Saudi Arabia; 2 Department of Pharmaceutics, Faculty of Pharmacy, Sinai University, Alarish, North Sinai 45511, Egypt; ali.nasr@su.edu.eg; 3 Department of Pharmacy Practice, Faculty of Pharmacy, University of Tabuk, Tabuk 471, Saudi Arabia; kgopal@ut.edu.sa; 4 Department of Pharmacology & Toxicology, Faculty of Pharmacy, Damanhour University, Damanhour 22511, Egypt; m.abdelhasseb@pharm.dmu.edu.eg; 5 Department of Pharmaceutics, Faculty of Pharmacy, The British University in Egypt, El-Sherouk city, Cairo 11837, Egypt; shady.swidan@bue.edu.eg; 6 The Center for Drug Research and Development (CDRD), Faculty of Pharmacy, The British University in Egypt, El-Sherouk city, Cairo 11837, Egypt; 7 Department of Pharmaceutics, Faculty of Pharmacy, Port Said University, Port Said 42511, Egypt

**Keywords:** carbamazepine, solid lipid nanoparticle, homogenization and ultra-sonication technique, pentylenetetrazole, anticonvulsant activity

## Abstract

**Objectives:** The study aimed to prepare carbamazepine in solid lipid nanoparticle form (CBZ-SLN) in order to enhance its anticonvulsant effect. **Method:** Eight formulations of CBZ-SLNs were prepared by homogenization and ultra-sonication techniques. **Results:** The prepared CBZ-SLN showed a high entrapment efficiency% (39.66 ± 2.42%–71.91 ± 1.21%), a small particle size (45.11 ± 6.72–760.7 ± 5.25 nm), and a negative zeta potential (from −21.5 ± 1.02 to −38.4 ± 1.32 mv). The in vitro release study showed the slow release of CBZ from SLNs compared to CBZ aqueous dispersion (*p* < 0.05). The infrared spectroscopy and the thermal analysis revealed the compatibility of the drug with other ingredients and the presence of drug in the more soluble amorphous estate, respectively. The in vivo study on mice revealed that the CBZ-SLN had a higher anticonvulsant efficacy than CBZ aqueous dispersion after a lethal and chronic dose of pentylenetetrazole (PTZ) (*p* < 0.05). The histopathological examination of the hippocampus revealed a decrease in the percentage of degeneration in mice treated with the CBZ-SLN compared to the PTZ and CBZ groups. **Conclusion:** CBZ can be formulated as SLN with higher anticonvulsant activity than free CBZ aqueous dispersion.

## 1. Introduction

Epilepsy is a disorder in the central nervous system (CNS) that is characterized by an increase in the number of electrical impulses that occurs in one focal locus of the brain and/or the entire brain, resulting in partial or generalized seizures [1].

The administration of antiepileptic drugs is mostly done via the oral or intravenous routes. Drug resistance at the late stage of therapy develops in about 40% of patients [2]. Drug resistance leads to uncontrolled seizures, a higher risk of brain damage, and increased mortality rates [3]. Patients with epilepsy suffer from emotional and behavioral changes, seizures, convulsions, muscular spasms, depression and, in some cases, unconsciousness [4]. Drugs used for the treatment of epilepsy have poor bioavailability and eventually become ineffective over the course of treatment due to drug resistance [5].

Epilepsy treatment is often complicated due to the poor release of the drug through the adjunctive blood–brain barrier, which may be overcome by the preparation of the drug as solid lipid nanoparticles. The ideal delivery systems of antiepileptic drugs are the ones which provide localized and controlled drug release to targeted sites in the brain, which helps to reduce drug-associated toxicities and enhances the efficacy of the drug [6,7].

Carbamazepine (CBZ) is an antiepileptic drug with a narrow therapeutic index, and it is poorly absorbed when taken by the oral route [8]. Its oral bioavailability is 75%, and its maximum achieved plasma concentration is about 4–8 h after administration [1]. Carbamazepine acts through the inactivation of sodium channels, making brain cells less excitable.

Solid lipid nanoparticles (SLNs) are considered an alternative to colloidal systems like liposome, noisome and polymeric nanoparticles [9]. SLNs consist of solid lipids, which act as a stabilizer when dispersed in water in the presence of a surfactant [10,11].

SLN is characterized by its small size (nano range), large surface area, and high drug loading capacity. In addition, it improves drug stability and has the ability to improve the oral bioavailability of poorly water-soluble drugs [12,13]. Gangurde and Kumar prepared lamotrigine solid lipid nanoparticles with a high efficacy for treatment of epilepsy [14]. Nair et al. prepared carbamazepine solid lipid nanoparticles to improve its therapeutic effect [2]. Kumar et al. prepared solid lipid nanoparticles of methylthioadenosine for the management of neurological conditions via oral delivery [15].

The objective of the current study was to prepare carbamazepine solid lipid nanoparticles (CBZ-SLNs) as a novel and alternative method for other colloidal dispersion carriers. The study aimed to prepare CBZ-SLNs with high entrapment efficiency%, small particle size, and high anticonvulsant activity compared to free CBZ.

## 2. The Experimental Part

### 2.1. Materials

Carbamazepine (CBZ) was obtained from the Novartis pharmaceutical company (Cairo, Egypt), Tween 80 from Sigma Chemical Company (Taufkirchen, Germany), poloxamer 188 was obtained from Sigma Aldrich (St. Louis, MO, USA), stearic acid was obtained from Hi-Media Laboratories Pvt. L-td. (Mumbai, India), glyceryl monostearate (GMS) was obtained from Sasol Germany GmbH (Witten, Germany), pentylenetetrazole was obtained from Sigma Aldrich (USA), sodium hydroxide and potassium hydrogen phosphate were obtained from Pure Lab (USA), uranyl acetate was obtained from Sigma Aldrich (USA), potassium bromide IR grade was obtained from Sigma Aldrich (USA), thiopental sodium was obtained from Egyptian International Pharmaceuticals Industries Co (EIPICO) (Cairo, Egypt), and saline was obtained from El Nasr company (Cairo, Egypt).

### 2.2. Methods

#### 2.2.1. The Preparation of the CBZ-SLN

Eight formulations of CBZ-SLNs were prepared by the modified hot high-shear homogenization ultra-sonication method. CBZ (10 mg) and the oily phase (100 g of stearic acid or glyceryl monostearate) were added in a small glass vial [4]. The content of vial was heated to 80 °C (which is above the melting points of both lipids) using a hot plate (Brandstead/Thermolyne, Swedesboro, NJ, USA) until the complete melting of the lipid. The aqueous phase was prepared by dissolving the surfactant (0.5% or 1% of Tween 80 or poloxamer 188) and heating to the same temperature as the oily phase (on a hot plate). The aqueous phase was added to the oily phase while keeping the temperature at 80 °C, and then they were homogenized using a Heidolph Silent Crusher^®^ homogenizer (Heidolph, Schwabach, Germany) at 20,000 rpm (5734 g) for 10 min [16]. A digital sonifier (Branson, Danbury, CT, USA) was used to sonicate the coarse emulsion for 15 min at a sonication power of 90% of maximum output. The sonicated dispersion was allowed to congeal for 2 hr at 4 °C, thus yielding SLNs [17].

#### 2.2.2. The Determination of Entrapment Efficiency% (EE%) of CBZ in the Prepared CBZ-SLN

The entrapment efficiency (EE)% of CBZ in the prepared CBZ-SLN was determined by an indirect method. The non-entrapped CBZ was separated from the entrapped one by centrifugation at 15,000 rpm (20629 g) for 45 min using centrifuge (Biofuge, primo Heraeus, Germany) [18]. The supernatant was analyzed using a UV spectrophotometer (Shimadzu, Tokyo, Japan) at 280 nm with an extinction coefficient of 9474 M^−1^ cm^−1^. The following equation was used to calculate the EE% of CBZ in the prepared CBZ-SLN [19].

Entrapment efficiency (%) = (Total CBZ − Free CBZ)/Total CBZ × 100

#### 2.2.3. The Determination of Particle Size, Zeta Potential, and the Polydispersity Index of the Prepared CBZ-SLN

All the prepared CBZ-SLNs were subjected to the measurement of particle size (ps), zeta potential (zp) and polydispersity index (PDI) using the dynamic light scattering (DLS) technique with a Zetasizer (Malvern Instruments Ltd., Malvern, UK) at 25 °C and a measuring angle of 90°. Each sample was diluted with an appropriate amount of distilled water before the analysis at a concentration of 1% [20].

#### 2.2.4. The In Vitro Release Study of CBZ from the Prepared CBZ-SLN

The release study of CBZ from the prepared CBZ-SLN and CBZ aqueous dispersion was conducted using Franz’s diffusion cell apparatus (Maharashtra, Mumbai, India). The apparatus consisted of seven Franz cells. In each cell, 2 mL of the prepared CBZ-SLN formulation or CBZ aqueous dispersion was added in the donor cell. The receptor cell was filled with 10 mL of a phosphate buffer (PH 7.4), and the diffusion medium was kept at 37 ± 1 °C and stirred at 100 rpm [4]. The donor and receptor cells were separated by a dialysis membrane (molecular weight cut off 12.000–14.000) through which the CBZ was released. Samples were withdrawn from the receptor cell at different time intervals (1, 2, 4, 6, 8, 10 and 12 h) and then analyzed by an ultraviolet spectrophotometer at 280 nm. Then, the amount of released CBZ was calculated. The experiment was done in triplicate, and the mean ± SD was calculated.

The permeation data were analyzed with the following mathematical models: zero-order kinetic, first-order kinetic, Korsmeyer–Peppas kinetic model, and Higuchi kinetic model [21]. The best mechanism for CBZ permeation was investigated.

The statistical analysis was conducted using a one-way analysis of variance (ANOVA). The Statistical Package for Social Science, version 16 (SPSS Software; SPSS Inc., Chicago, IL, USA) was employed for data analysis. The differences were considered to be significant if *p* < 0.05.

#### 2.2.5. The Optimization of Formulation Variables

The choice of the best formulation, which was intended to complete the in vivo study, was selected based on a high entrapment efficiency%, small particle size, a high zeta potential to avoid the aggregation of nanoparticles, and a high drug release after 12 h (Cumulative amount of drug released after 12 h, Q12) [22].

#### 2.2.6. The Morphological Examination of the Best Formulation of the CBZ-SLN

##### Transmission Electron Microscope (TEM)

The surface morphology of the best CBZ-SLN formulation was examined by a transmission electron microscope (JTEM model 1010, JEOL^®^, Tokyo, Japan). A sample of the best CBZ-SLN formulation was diluted with distilled water followed by the application of one drop in a collodion-coated copper grid. After the sample was dried, it was stained by a uranyl acetate solution and examined by TEM after drying at an acceleration voltage of 100 kV [23,24].

##### Scanning Electron Microscope (SEM)

The surface morphology of the optimized formulation of the CBZ-SLN (F8), which was prepared by GMS and poloxamer 188, was evaluated using a scanning electron microscope (JEOL, JSM 50A, Tokyo, Japan). One drop of the sample was spread on an aluminum stub and then allowed to dry at 25 ± 1 °C. The dried sample was coated with thin layer of platinum, and the image was captured by SEM at 30 Kv [21].

#### 2.2.7. The Fourier-Transform Infrared Spectroscopy (FT-IR)

Fourier-transform infrared spectroscopy (FT-IR) was conducted to ensure that there was no intermolecular interaction between CBZ and other SLN ingredients [25]. Pure CBZ, GMS, Stearic acid, and SLNs were prepared with GMS, and SLNs prepared with stearic acidwere subjected to FT-IR using a Shimadzu 435 U-O4 IR spectrometer (Japan). Each of the previous samples was compressed into a disc in presence of potassium bromide, and they were then subjected to scanning from 4000 to 400 cm^−1^.

#### 2.2.8. The Differential Scanning Calorimetry (DSC)

The lyophilized samples of pure CBZ, GMS, Stearic acid, and SLNs prepared with GMS, as well as SLNs prepared with stearic acid, were evaluated for their thermotropic properties and phase transition behavior using Shimadzu DSC-50 (Japan). Samples of about 3 mg were heated and sealed in an aluminum pan ranging from 25 to 250 °C at a rate of 10 °C/min under nitrogen gas flow [2]. An empty aluminum pan was used as a standard.

#### 2.2.9. Pharmacological Activity

##### Animals

The animals were purchased from the modern veterinary office (Cairo, Egypt). Male albino mice (8–10 weeks old) weighing 25–28 g at the time of the experiment were used and were housed 6 mice per cage under a normal light/dark cycle with free access to food and water ad libitum. All experimental protocols were approved by the research ethics committee at the Faculty of Pharmacy, Damanhour University (approval number 419PO15), Elbehera, Egypt, and all efforts were made to decrease and eliminate animal suffering.

##### Time to Death Test

Twenty four male albino mice were used in this test and were randomly divided into 4 groups: (1) the saline control group (administrated water oral and saline intraperitoneal (i.p.)), (2) the pentylenetetrazole (PTZ) control group that received PTZ (70 mg/kg, i.p.) after 45 min from oral water administration, (3) the CBZ group that received CBZ aqueous dispersion (50 mg/kg, p.o.) 45 min before PTZ injection (70 mg/kg, i.p), and (4) the CBZ-SLN group that received the CBZ-SLN formulation (50 mg/kg, p.o.) 45 min before PTZ injection (70 mg/kg, i.p.). This experiment was designed to compare the anticonvulsant effect of the prepared CBZ-SLN and an aqueous dispersion of free CBZ. This comparison was made by measuring the time to death in mice after a lethal dose of PTZ [26].

##### Seizure Activity Evaluation in PTZ-Kindled Mice

Twenty four male albino mice were used in this study and were randomly divided into 4 groups; each group consisted of 6 mice: (1) the saline control group (administrated water orally and saline i.p.), (2) the PTZ control group that received PTZ (35 mg/kg, i.p.) after 45 min from oral water administration, (3) the CBZ group that received CBZ dispersed in water (50 mg/kg, p.o.) 45 min before PTZ injection (35 mg/kg, i.p), and (4) the CBZ-SLN group that received the CBZ-SLN formulation (50 mg/kg, p.o.) 45 min before PTZ injection (35 mg/kg, i.p.).

Each mouse was observed for convulsion behavior for 30 min after the injection of a chronic dose of PTZ [27]. Each mouse was given a score according to the severity of seizures based on Racine rating scale for seizure evaluation [28]. Mice were scored according to the following criteria: (0): if mice showed no seizure; (1): if mice showed immovability, eye closing, ear twitching, and facial clonus; (2): if mice showed head nodding associated with more severe facial clonus; (3): if mice showed the clonus of 1 forelimb; (3.5): if mice showed bilateral forelimb clonus with no rearing; (4): if the mice showed bilateral forelimb clonus with rearing; (4.5): if the mice were dropping on a side (without rearing), loss of righting reflex accompanied by general tonic clonic seizures; and (5): if mice were rearing and falling on back convoyed by general tonic clonic seizures. After the last PTZ injection, the mean of fifteen seizure scores was calculated for each group and then compared [29].

##### Tissue Preparation:

After the last PTZ injection by 24 h, mice were anesthetized using thiopental sodium (50 mg/kg) and then were sacrificed by cervical dislocation. The brains of the mice were isolated, and the right hemisphere was fixed for 24 h in 10% paraformaldehyde solution and subjected to staining for histopathological examination [30].

##### The Preparation of Histopathological Slides

The brain tissues were cut until the hippocampus appeared. Tissues were subjected to washing, dehydration with ascending graded series of ethanol, and then embedding in pure paraffin wax in disposable tissue molds. From the paraffin block of each mouse, 2 sections were prepared in a thickness of 4 μm using microtome (Leitz 1512, Wetzlar, Germany). The microscopic slides were stained with hematoxylin plus eosin (H and E) or 1% and Cresyl violet stain (Nissil stain), washed in phosphate buffer salin (PBS), and cover-slipped with a water-based mounting medium.

##### The Histopathological Examination of Cell Degeneration in the Hippocampus Region

Cell degeneration in the region of hippocampus was evaluated by H and E staining after the administration of PTZ. A light microscope (Olympus^®^, Japan) was used for the examination of the hippocampus, where 5 regularly spaced sections covering the hippocampus were imaged and analyzed for each mouse [31]. Each section was examined for the count of the degenerative cells which were characterized by acidophilic cytoplasm and darkly stained or heterochromatic pyknotic nuclei [32]. The determination of the total number of degenerative cells in the hippocampus was done by calculating the sum of cell counts from the 5 selected sections followed by the calculation of the mean for each mouse, and then the calculation of it as a percent of the total cell number [33]. All H and E stained slides were captured at the original magnification 200× (Objective 20×) with a UIS optical system (Universal Infinity System, Olympus^®^, Japan).

##### The Histopathological Detection of Surviving Cell in Hippocampus by Cresyl Violet Staining

The Cresyl violet stain was used to mark the Nissl bodies. These Nissl substances are bundles of ribosomes related to the endoplasmic reticulum membrane of the neurons [34]. The hippocampus was selected to be stained because it is the most sensitive area of the brain to toxic insults and is critical for controlling epileptic seizures.

Surviving neuron morphology was detected in 5 regularly spaced sections covering the whole surface of the hippocampus. The surviving cells were recognized by a distinct and complete neural cell membrane in addition to a visible rounded nucleus [35]. The average of the surviving neurons in the 5 selected sections was calculated for each mouse in each group and calculated as a percent of the total cell number [32,36]. All Cresyl violet stained slides were captured at the original magnification of 400× (Objective 40×) with a UIS optical system (Universal Infinity System, Olympus^®^, Japan). All the histopathological examinations were blindly conducted by two pathologists unaware of the study groups.

##### Statistical Analyses

Data were represented as a mean ± SD and was analyzed using an ANOVA followed by Bonferroni’s post hoc. The Statistical Package for Social Science, version 16 (SPSS Software; SPSS Inc., Chicago, IL, USA) was used for data analysis. The significance level was set at *p* < 0.05.

## 3. Results and Discussion

### 3.1. The Preparation of the CBZ-SLN

Eight formulations of CBZ-SLNs were prepared by the modified hot high-shear homogenization ultra-sonication method (F1–F8) [15]. The composition of the prepared formulations is listed in Table 1. All the prepared CBZ-SLN formulations were evaluated for EE%, particle size analysis, PDI, zeta potential, and in vitro release of CBZ to determine the best formulation.

### 3.2. The Entrapment Efficiency% (EE%) of CBZ in the Prepared CBZ-SLN

As shown in Table 2, the EE% of CBZ in the prepared CBZ-SLN ranged from 39.66 ± 2.42% for F1 to 71.91 ± 1.20% for F8.

#### 3.2.1. The Effect of Lipid Type on the EE% of CBZ in the Prepared CBZ-SLN

**Figure 1:**
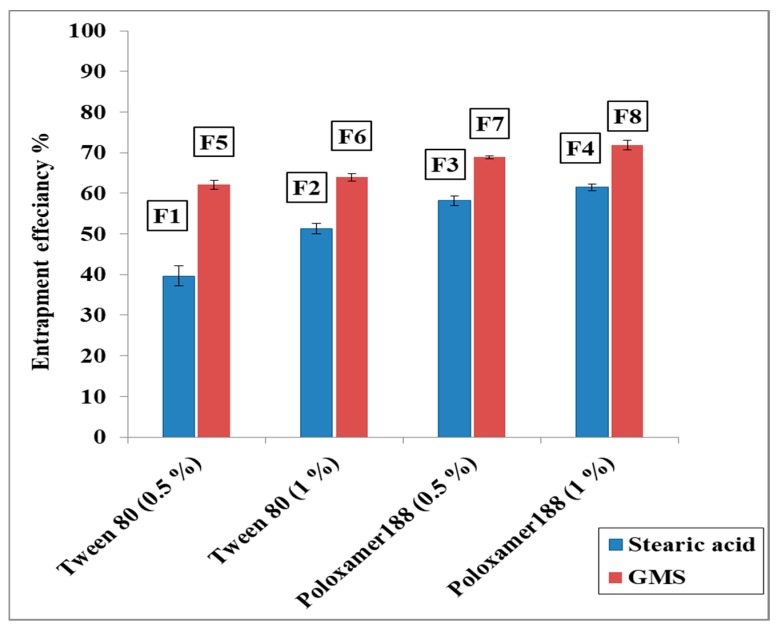
The effect of lipid type and surfactant concentration on the entrapment efficiency% of carbamazepine in the prepared carbamazepine solid lipid nanoparticles formulations.

As shown by Figure 1, it was indicated that EE% of CBZ is higher in case of GMS than in case of stearic acid. The EE% of CBZ for formulations prepared with GMS was 62.08 ± 1.05–71.91 ± 1.20%, while for formulation prepared with stearic acid was 39.66 ± 2.42–61.49 ± 0.85%. This may be attributed to the long carbon chain of GMS (C 21), which resulted in a less ordered solid lipid nanoparticle structure and offered great loading space to encapsulate more CBZ drug molecules [4]. These results were in accordance with the study of Priyanka et al. [21], in which the EE% of montelukast sodium in the prepared SLNs is higher in the case of GMS than in the case of stearic acid.

#### 3.2.2. The Effect of Surfactant Type and Concentration on the EE% of CBZ in the Prepared CBZ-SLN

As shown in Figure 1, it was revealed that the EE% of CBZ was higher in case of poloxamer 188 than in case of Tween 80. The EE% of CBZ for formulations prepared with poloxamer 188 was 58.16 ± 1.15–71.91 ± 1.20%, while for formulation prepared with Tween 80, it was 39.66 ± 2.42–63.95 ± 0.89%. This may be due to the higher HLB value (Hydrophilic Lipophilic Balance) of poloxamer 188 than that of Tween 80 [37]. This was similar to the result obtained by Ekambaram et al. [37], who found that the entrapment efficiency of various Ramipril SLNs stabilized with different nonionic surfactants, and the EE% in case of poloxamer 188 was greater than that of Tween 80 and Span 20.

Additionally, the EE% of CBZ was higher in the case of 1% surfactant than in the case of 0.5% for both Tween 80 and poloxamer 188. This may be due to the increase in the solubility of the drug in the lipid and the increase in the steric stability of the lipid, resulting in an increase of the concentration of the surfactant [36]. These results are in good agreement with those of Abdelbary et al. [4] who found that the EE% of diazepam in the prepared SLN increased by increasing the surfactant concentration.

### 3.3. The Particle Size, Zeta potential, and Polydispersity Index of the Prepared CBZ-SLN

As illustrated by Table 2, the particle size of the prepared CBZ-SLN was small in the nano range (45.11 ± 6.72–760.7 ± 5.25 nm). It was revealed that the particle size of the prepared CBZ-SLN differed according to the type of lipid and the type and concentration of used surfactants.

#### 3.3.1. The Effect of Lipid Type on the Particle Size

**Figure 2:**
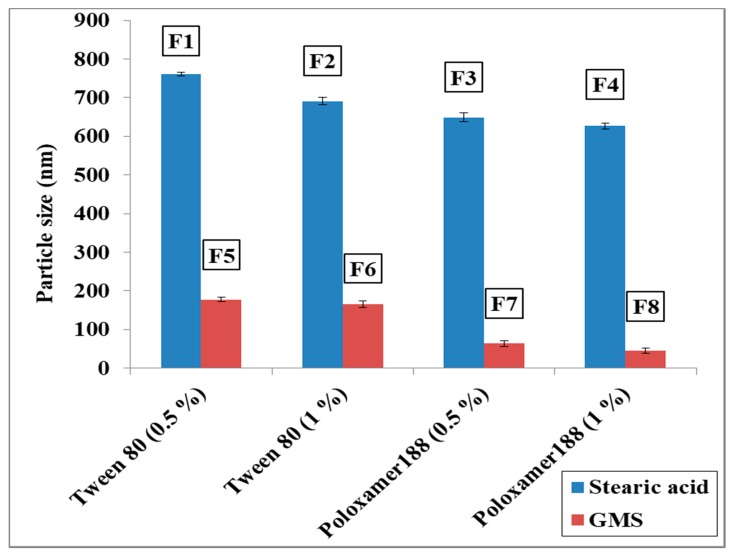
The effect of lipid type and surfactant concentration on the particle size of the prepared carbamazepine solid lipid nanoparticles formulations.

The particle size of the prepared CBZ-SLN formulations, which were prepared using GMS, was smaller than that of those prepared using stearic acid (see Figure 2). This may be attributed to the fact that stearic acid has a higher melting point than GMS, which results in a slower lipid crystallization during preparation. Accordingly, this leads to increasing the particle size [21]. Additionally, it has been proposed that GMS acts as a surfactant and a lipid matrix which allows for the emulsification and preparation of small CBZ-SLNs. This result was in agreement with the results obtained by the study of Abdelbary et al. [4], in which the particle size of a diazepam SLN prepared by stearic acid was larger than that prepared by GMS.

#### 3.3.2. The Effect of Surfactant Type and Concentration on the Particle Size

As shown by Figure 2, the particle size of the prepared CBZ-SLN formulations, which were prepared by poloxamer 188, was smaller than that of those prepared by Tween 80. This may be attributed to the high HLB value of poloxamer 188.

Additionally, the particle size of the prepared CBZ-SLN decreased as the concentration of the surfactant increased from 0.5% to 1%, irrespective of the type of surfactant. This may be attributed to the decrease in the interfacial tension between the aqueous and lipid phases during preparation as the surfactant concentration increased, resulting in a small droplet of lipid in the internal phase of the emulsion, which, after cooling, can lead to the preparation of small SLNs [38].

Additionally, the increase in surfactant concentration leads to an increase in the stearic stability of the prepared CBZ-SLN, which prevents the aggregation of a prepared SLN [38].

As shown by Table 2, all the prepared CBZ-SLNs had negative zeta potentials that ranged from −21.5 ± 1.02 to −38.4 ± 1.32 mv. All the prepared CBZ-SLNs exhibited negative zeta potentials with high values, which indicates a high stability of the prepared CBZ-SLNs due to a lesser tendency for aggregation [2]. These results are similar to those obtained by Nair et al. [2], who reported that the prepared carbamazepine solid lipid nanoparticles have a negative zeta potential with high values.

As shown by Table 2, the values of the PDI for all the prepared CBZ-SLNs ranged from 0.196 ± 0.05 to 0.419 ± 0.02, which is less than 0.5. This indicates a narrow size distribution and a homogenous distribution [20].

### 3.4. The In Vitro Release Study of CBZ from the Prepared CBZ-SLN

**Figure 3:**
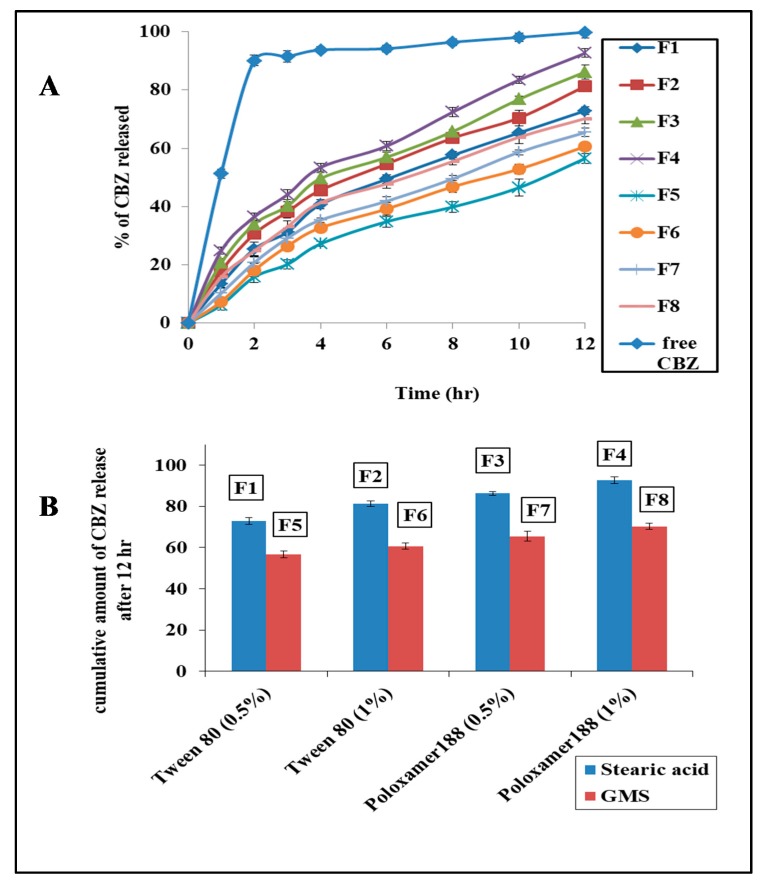
The in vitro release study. (**A**) The release profile of carbamazepine from the prepared carbamazepine solid lipid nanoparticles in comparison to free carbamazepine aqueous dispersion, and (**B**) the effect of lipid type and surfactant concentration on the in vitro release of carbamazepine.

The results of the in vitro release study of all the prepared CBZ-SLNs (F1–F8) and the free CBZ are shown by Figure 3A.

It was shown that the release of CBZ from all the prepared CBZ-SLNs was much slower than its release from the free CBZ suspension (*p* < 0.05). These results indicate the availability of the sink condition for the release of CBZ, and there is no limitation for drug release through the dialysis membrane [4].

For all the prepared CBZ-SLNs, it was shown that the release was fast at first, which may be due to the drug absorption on the surface of the prepared SLNs. Then, the release became more sustained due to the diffusion of the drug from SLNs to the diffusion medium [39,40].

#### 3.4.1. The Effect of Lipid Type on Drug Release

As shown by Figure 3B, the drug release from SLNs prepared by GMS was more sustained than that of those prepared by stearic acid. This may be attributed to the long carbon chain of GMS (C 21), which resulted in less ordered lipid crystals with less drug expulsion and a slower drug release [4].

#### 3.4.2. The Effect of Surfactant Type and Concentration on Drug Release

The drug release from the SLNs prepared by poloxamer 188 was faster than that of those prepared by Tween, and the release was more sustained in case of 0.5% surfactant than in case of the 1% surfactant. This may be due to the fact that the smaller the particle size, the higher the surface area and the smaller the diffusion distances, which resulted in higher dissolution and drug release; Figure 3B [39,41].

#### 3.4.3. Release Kinetics

The release kinetics for all the prepared CBZ-SLNs was evaluated to determine the release behavior of CBZ from the prepared SLNs. The release data were analyzed with zero-order kinetic, first-order kinetic, and Korsmeyer–Peppas kinetic models, as well as the Higuchi kinetic model. As shown by Table 3, it was revealed that the release data from SLNs fit to Higuchi kinetic model with the highest (r) value, while for free CBZ suspension, the release data fit the first-order kinetic model. These results were found to be in agreement with those obtained by Venkateswarlu and Manjunath, who found that the release data for clozapine from the prepared SLNs fit to the Higuchi diffusion model [3].

### 3.5. The Selection of the Optimized Formulation

After ranking the prepared CBZ-SLN formulation based on a high EE%, a small particle size, a high zeta potential, and a fast drug release, it was shown that F8 was the best one and therefore the one chosen to complete the in vivo study. The EE% was 71.91 ± 1.20%, the particle size was 45.11 ± 6.72 nm, the zeta potential (ZP) was -33.3 ± 1.45 mv, and the Q12 was 70.23 ± 1.48% [42].

### 3.6. The Surface Morphology of the CBZ-SLN

**Figure 4:**
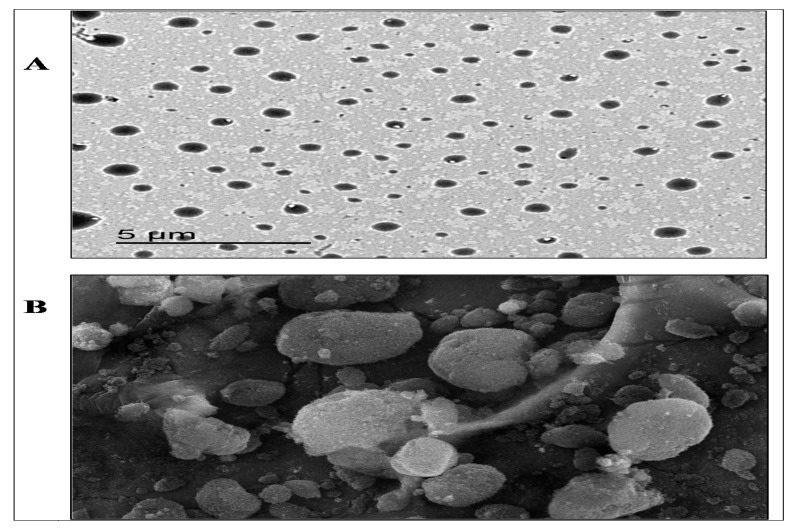
The surface morphology of the optimized formulation of the carbamazepine solid lipid nanoparticles (F8). (**A**) Transmission electron microscopy image and (**B**) scanning electron microscope image (×500).

As depicted by Figure 4A, the TEM images of the optimized formulation of the CBZ-SLN (F8) revealed that the prepared CBZ-SLN possessed a uniform spherical shape with nanometer size range [11]. As shown in Figure 4B, the SEM photograph of the optimized formulation of the CBZ-SLN (F8) was spherical with a smooth surface.

### 3.7. FT-IR Spectroscopy

**Figure 5:**
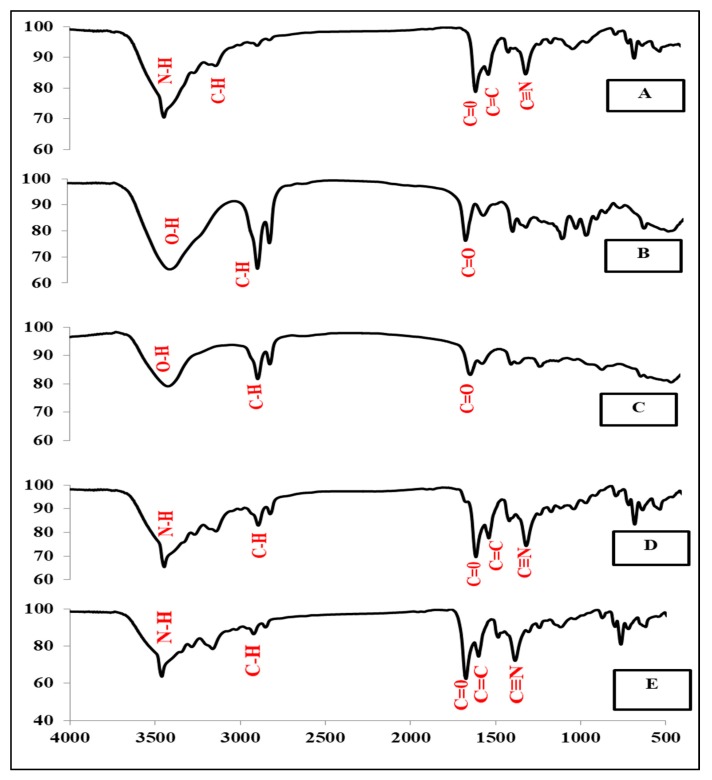
Infrared spectra of (**A**) pure carbamazepine, (**B**) glyceryl monostearate, (**C**) stearic acid, (**D**) solid lipid nanoparticle prepared with glyceryl monostearate, and (**E**) solid lipid nanoparticle prepared with stearic acid.

FT-IR spectroscopy was employed to investigate the interactions between the lipid, the drug and other excipients, as shown by Figure 5. The FT-IR spectra of the pure drug (CBZ) showed a characteristic peak of an N–H stretch at 3459.67 cm^−1^, a C=0 stretch at 1672.95 cm^−1^, an aromatic C–H stretch at 3163.65 cm^−1^, a C=C stretch at 1600.63 cm^−1^ and C≡N at 1385.6 cm^−1^, see Figure 5A. The IR-spectrum of CBZ was similar to the results obtained by Nair et al. [2], which showed the same peaks.

The IR-spectra of GMS, as shown by Figure 5B showed characteristic peaks of broad O–H stretching at 3419.17 cm^−1^, C–H stretching at 2919.7 cm^−1^, and C=O stretching at 1735.62 cm^−1^ [21].

As shown by Figure 5C, the IR-spectra of stearic acid showed characteristic peaks of broad O–H stretching at 3435.56 cm^−1^, C–H stretching at 2919.7 cm^−1^, and C=O stretching at 1698.98 cm^−1^. These results are in agreement with those obtained by Garg et al. [43].

The IR-spectra of the CBZ-SLN prepared with GMS and stearic acid showed all the characteristic peaks of CBZ and lipids, as shown by Figure 5D,E, respectively.

The presence of the characteristic peaks of CBZ and lipids in the SLN formulation proves that the drug remained intact without any interactions [2,21].

### 3.8. The Thermal Analysis of the CBZ-SLN

Thermal analysis is an important method that gives an indication of the melting point and recrystallization behavior of crystalline substances.

**Figure 6:**
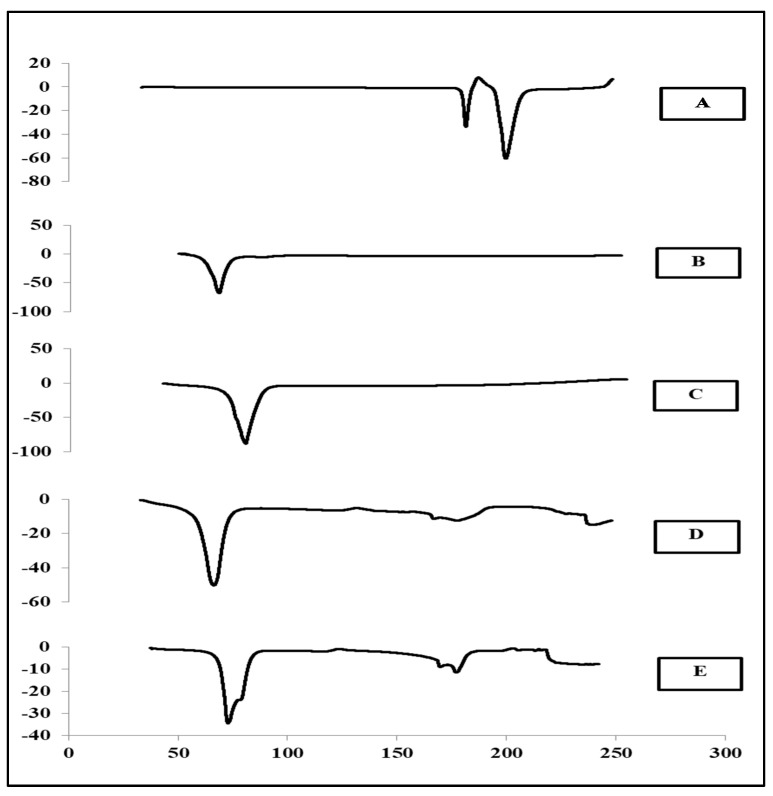
The thermal analysis of (**A**) pure carbamazepine, (**B**) glyceryl monostearate, (**C**) stearic acid, (**D**) solid lipid nanoparticle prepared with glyceryl monostearate, and (**E**) solid lipid nanoparticle prepared with stearic acid.

As shown by Figure 6A, the differential scanning calorimetry (DSC) thermogram of the pure CBZ exhibited two endothermic peaks—the first at 177.05 °C, which was due to the beta form of the CBZ moving to the alfa form, and the second endothermic peak at 194.88 °C, which indicates the melting point of CBZ [10]. This DSC thermogram of CBZ is similar to previous studies carried out by Nair et al., Gavini et al., and Rustichelli et al. [2,8,44].

As shown by Figure 6B,C, the DSC thermogram of the two lipids used in this study (GMS and stearic acid) exhibited sharp endothermic peaks of the melting point at 65.49 and 76.56 °C, respectively [21].

For the CBZ-SLN prepared by GMS as a lipid, the DSC thermogram showed only one endothermic peak of GMS at 64.56 °C; see Figure 6D. Additionally, as illustrated by Figure 6E, the thermal analysis of the CBZ-SLN prepared by stearic acid as a lipid exhibited only one melting endothermic peak of stearic acid at 74.09 °C. The disappearance of the CBZ peak indicates the dispersion of the drug in the lipid matrix in a more soluble amorphous state [10,38]. The same results were obtained by Scioli et al. [40], who found that the endothermic peak of CBZ disappeared in the prepared SLN, and the drug converted from the crystalline state into the amorphous state.

### 3.9. The Pharmacological Activity of the CBZ-SLN

#### 3.9.1. The Evaluation of the Anticonvulsant Activity of the CBZ-SLN by Time to Death Test

The ability of the CBZ-SLN to prolong the time to death after a lethal dose of PTZ (70 mg/kg, i.p.) was used to evaluate its anticonvulsant activity and compare it with the free CBZ aqueous dispersion [25].

**Figure 7:**
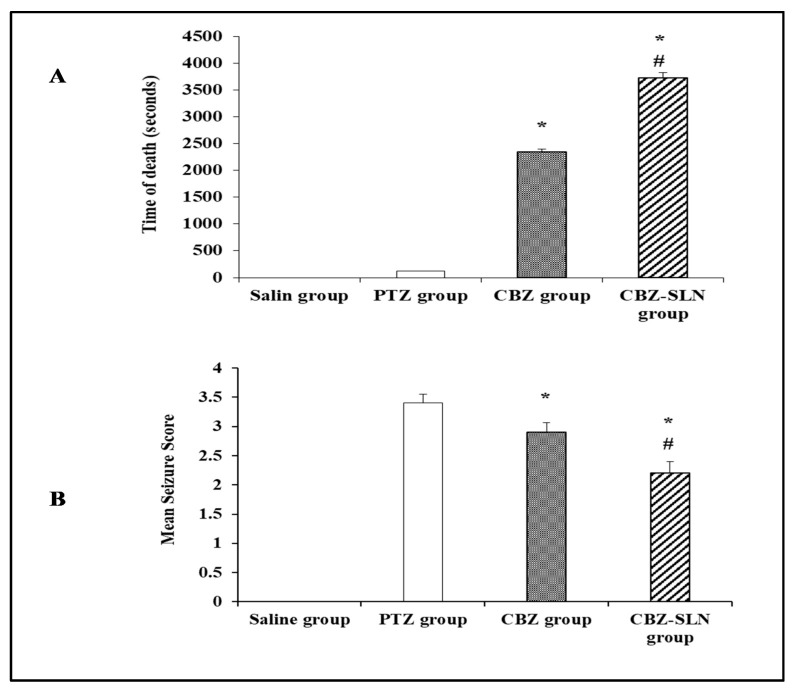
The pharmacological activity of carbamazepine solid lipid nanoparticles; (**A**) the effect of free carbamazepine and carbamazepine solid lipid nanoparticles (F8) on the time to death after the administration of a lethal dose of pentylenetetrazole (PTZ), and (**B**) the effect of free carbamazepine and carbamazepine solid lipid nanoparticles (F8) on mean seizure scores in pentylenetetrazole (PTZ)-kindled mice. Data are presented as mean ± SD (n = 6) and were analyzed using an ANOVA followed by Bonferroni’s post hoc test at *p* < 0.05. * Compared to the PTZ group, # compared to the CBZ group.

As shown by Figure 7A, it was found that the prepared CBZ-SLN successfully antagonized the lethality of PTZ. The results illustrated by Figure 7A showed that the time to death calculated for the CBZ-SLN group was significantly higher than that calculated for the CBZ and PTZ groups. The time to death for the PTZ, CBZ, and CBZ-SLN groups was found to be 120 ± 2.48, 2340 ± 140.71, and 3720 ± 244.95 s, respectively, *p* < 0.05. The prolonged anticonvulsant effect of the prepared CBZ-SLN formulation in comparison with the free CBZ aqueous dispersion may be attributed to the sustained drug release from the SLN and the enhanced absorption of the CBZ-SLN [11,40].

#### 3.9.2. The Effect of the CBZ-SLN on Seizure Score in Mice Kindled with PTZ

In this study, 15 repetitive injections of PTZ were used to achieve kindling in mice. As shown by Figure 7B, the mice in the PTZ group showed an increase in the mean seizure score with repetitive PTZ injections. The final seizure score for the PTZ group was 3.4 ± 0.38, while it was 0 ± 0 (*p* < 0.05) for the saline group. The final seizure scores for the CBZ and CBZ-SLN groups were 2.9 ± 0.41 and 2.2 ± 0.49, respectively, which re significantly lower than that of the PTZ group. The percentage of reduction in the final seizure score for the CBZ aqueous dispersion group was 14.71%, and it was 35.29% for the CBZ-SLN group. The percentage of reduction in the final seizure score for the CBZ-SLN group was significantly higher than that for the CBZ group, *p* < 0.05. These results may be owed to the nano size and different characters of nanoparticles compared to the conventional form of the drug. The nano size of nanoparticles has the ability to alter their physicochemical properties and allow for increased permeability through biological membranes [45].

#### 3.9.3. The Effect of Repeated Administration of the CBZ-SLN on the Percentage of Degenerative Cells in the Hippocampal Sections Stained with H and E

**Figure 8:**
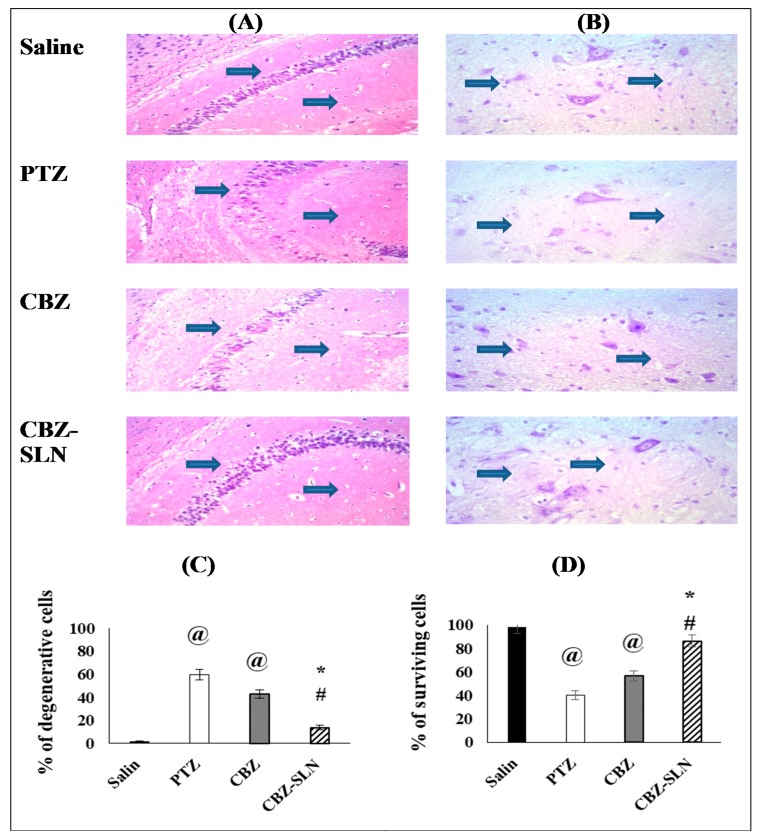
The effect of all pharmacological treatments in the photographic pictures of the hippocampus sections. (**A**) Histopathological pictures stained with hematoxylin plus eosin, (**B**) photographic pictures of hippocampus sections stained with Cresyl violet, (**C**) the percentage of degenerative cells in the hippocampal area of the different groups, and (**D**) the percentage of surviving cells in the hippocampal area of the different groups. Data are presented as mean ± SEM and were analyzed using a one-way ANOVA followed by Bonferroni’s post-hoc test at *p* < 0.05. @ compared to the saline group, * Compared to the PTZ group, # compared to the CBZ group.

Figure 8A shows the photographic pictures of the hippocampal region for all the groups under study. The saline group showed preserved orderly arranged neurons in multiple layers, indicating vesicular nuclei and eosinophilic cytoplasm with fibrillary background and no degenerative changes. The PTZ-kindled group showed a marked loss of an orderly layered arrangement of neurons, with many degenerated neurons showing shrinkage, decreased layers and dispersion [30]. The CBZ group showed a moderate improvement, with a mild distortion of neurons arrangement with few degenerated neurons showing decreased layer thickness and dispersion. The CBZ-SLN group showed a marked improvement and regularly arranged neurons.

As shown by Figure 8C, the statistical analysis proved that there was a significant increase in the percentage of degenerative cell in the PTZ group compared to the saline group (*p* < 0.05), while for the CBZ and CBZ-SLN groups, there was a significant reduction in the percentage of degenerative cells compared to the PTZ group (*p* < 0.05). It was shown that the reduction in the percentage of degenerative cells attained by the CBZ-SLN group was significantly higher than that achieved by the CBZ group (*p* < 0.05).

These results may be attributed to the increased retention of the nanoparticles in the brain blood capillaries and the effect of the surfactant in the opening of the tight junctions between the brain endothelial cells, thereby enhancing the in vivo activity [38].

#### 3.9.4. The Effect of the CBZ-SLN on the Survival of Neurons in the Hippocampus of PTZ-Kindled Mice

Figure 8B shows the photographic pictures of the hippocampus of the groups under study stained by Cresyl violet staining. The saline group showed basal ganglia with abundant cytoplasm and preserved granules. The PTZ-kindled group showed smudged nuclei of the basal ganglia with the distortion of granules.

The CBZ group showed a slightly distorted granules, and the CBZ-SLN group showed regular basal ganglia with prominent granules.

From the histopathological examination, it was shown that the percentage of surviving cells in the saline group was 100%; in the PTZ group, it was 40.27%; in the CBZ group, it was 56.89%, and in the CBZ-SLN group, it was 86.30%, see Figure 8D.

The statistical analysis showed that the surviving neurons percentage in the PTZ group was significantly lower than that of the saline group, while the percentages of surviving cells in the CBZ-SLN and CBZ groups were significantly higher than that of the PTZ group. Additionally, it was shown that the percentage of living neurons in the CBZ-SLN group was significantly higher than that in case of the CBZ group.

Similar results were obtained by Leyva-Gómez et al. [11], who found that nanoparticle formulation improved the anticonvulsant effect of clonazepam on the PTZ-induced seizures compared to the free clonazepam.

These results may be attributed to the high absorption of the CBZ-SLN through the blood brain barrier by adsorption of the CBZ-SLN into the capillaries walls followed by a release of drug from this site to the brain or by adhesion of the CBZ-SLN to the membrane of the endothelial cells, followed by endocytosis of the endothelial cells and the drug release (passive diffusion) of these cells for brain delivery [46,47,48,49,50].

## 4. Conclusions

Through this study, the authors concluded that CBZ could be formulated in the form of SLNs using solid lipids (glyceryl monostearate or stearic acid) and emulsifier (Tween 80 or poloxamer 188). The prepared CBZ-SLNs had a high entrapment efficiency% that ranged from 39.66 ± 2.42% to 71.91 ± 1.21%, a small particle size with a range of 45.11 ± 6.72–760.7 ± 5.25 nm, a high negative zeta potential ranged from −21.5 ± 1.02 to −38.4 ± 1.32 mv, and a controlled drug release as compared to the release of drug from free aqueous dispersion. Additionally, the authors concluded that the solid lipid nanoparticles have the ability to increases the anticonvulsant activity of the CBZ, which is represented in the prolongation of the time to death after a lethal dose of PTZ in mice and a decrease the seizure score after the administration of a chronic dose of PTZ in mice.

## Figures and Tables

**Table 1:** The composition of carbamazepine solid lipid nanoparticles formulations.

Formulation No.	Drug (mg)	Type of Lipid	Type of Surfactant	Surfactant% (*w*/*v*)	Total Volume (mL)
**F1**	10	Stearic acid	Tween 80	0.5% (100 mg)	20
**F2**	10	Stearic acid	Tween 80	1% (200 mg)	20
**F3**	10	Stearic acid	Poloxamer 188	0.5% (100 mg)	20
**F4**	10	Stearic acid	Poloxamer 188	1% (200 mg)	20
**F5**	10	GMS	Tween 80	0.5% (100 mg)	20
**F6**	10	GMS	Tween 80	1% (200 mg)	20
**F7**	10	GMS	Poloxamer 188	0.5% (100 mg)	20
**F8**	10	GMS	Poloxamer 188	1% (200 mg)	20

GMS: glyceryl monostearate.

**Table 2:** The entrapment efficiency%, particle size, zeta potential, and the polydispersity index of the prepared carbamazepine solid lipid nanoparticles formulations.

Formulation No.	Entrapment Efficiency% EE (%)	Particle Size (nm)	Zeta Potential (mv)	Polydispersity Index (PDI)
**F1**	39.66 ± 2.42	760.7 ± 5.25	−25.1 ± 0.98	0.234 ± 0.08
**F2**	51.22 ± 1.28	691.1 ± 9.02	−27.6 ± 0.75	0.196 ± 0.05
**F3**	58.16 ± 1.15	648.7 ± 11.54	−27.7 ± 0.85	0.277 ± 0.01
**F4**	61.49 ± 0.85	626.5 ± 7.25	−21.5 ± 1.02	0.217 ± 0.04
**F5**	62.08 ± 1.05	177.8 ± 6.24	−32.5 ± 1.14	0.394 ± 0.07
**F6**	63.95 ± 0.89	165.0 ± 8.21	−38.4 ± 1.32	0.419 ± 0.02
**F7**	68.88 ± 0.37	63.30 ± 8.27	−30.1 ± 0.58	0.318 ± 0.04
**F8**	71.91 ± 1.20	45.11 ± 6.72	−33.3 ± 1.45	0.277 ± 0.03

**Table 3:** The calculated correlation coefficients for the in vitro release of carbamazepine employing different kinetic orders or systems.

Formulation No.	Correlation Coefficient (r)					
	Zero	First	Second	Diffusion	H-C	B-L
**F1**	0.983	−0.998	0.985	**0.999**	0.996	0.994
**F2**	0.984	−0.991	0.945	**0.998**	0.995	0.984
**F3**	0.987	−0.985	0.919	**0.997**	0.993	0.978
**F4**	0.991	−0.973	0.861	**0.998**	0.991	0.974
**F5**	0.987	−0.993	0.983	**0.996**	0.993	0.976
**F6**	0.977	−0.994	0.993	**0.996**	0.990	0.992
**F7**	0.983	−0.996	0.988	**0.997**	0.994	0.989
**F8**	0.982	−0.997	0.988	**0.998**	0.995	0.994
**Free CBZ**	0.652	**−0.910**	0.679	0.737	0.880	0.866
